# Evaluation of the effectiveness of photodynamic therapy for the endodontic treatment of primary teeth: study protocol for a randomized controlled clinical trial

**DOI:** 10.1186/s13063-015-1086-2

**Published:** 2015-12-03

**Authors:** Ana Carolina Costa da Mota, Marcela Leticia Leal Gonçalves, Carolina Bortoletto, Silvia Regina Olivan, Monica Salgueiro, Camila Godoy, Olga Maria Altavista, Marcelo Mendes Pinto, Anna Carolina Horliana, Lara J. Motta, Sandra Kalil Bussadori

**Affiliations:** Biofotonics Applied to Science Health Post Graduation Program Nove de Julho University, Rua Vergueiro, 249 - Liberdade CEP 0154001, São Paulo, SP Brasil

**Keywords:** Photodynamic Therapy, Endodontic treatment, Primary tooth

## Abstract

**Background:**

The elimination of pathogenic microorganisms from the root canal system is one of the major steps required for successful endodontic treatment. The aim of the proposed study is to conduct a randomized, controlled, clinical trial for the clinical and radiographic evaluation of the effectiveness of photodynamic therapy during the endodontic treatment of primary teeth.

**Methods:**

Thirty primary anterior teeth in children aged 3 to 6 years old will be randomly divided into 2 groups: a control group, which will receive conventional treatment, and an experimental group, which will be subjected to photodynamic therapy. Microbiological evaluations will be performed before and after endodontic treatment. Moreover, clinical and radiographic evaluations will be performed on the day of treatment as well as 1, 3 and 6 months after treatment. Comparisons will be made of the two study groups. The data will be tabulated and presented in a descriptive, analytical fashion. Depending on the distribution (normal or non-normal), either the *t* test, ANOVA or the Mann-Whitney test will be used for analysis of the variables. The Wilcoxon test will be used for comparisons before and after treatment. *P* values < 0.05 (95 % significance level) will be considered indicative of statistically significant differences.

**Discussion:**

As successful endodontic treatment is directly related to intra-canal bacterial disinfection and consideringthe difficult task of endodontic treatment in primary teeth, often due to difficulties in controlling youngchildren, the internal anatomy of root canals and root resorption, the alternative of using PDT is a painless,easy-to-administer method that does not lead to microbial resistance and can assist in the achievement ofsuccessful endodontic treatment in primary teeth by eliminating the pain children can experience due to retreatmentas well as premature tooth loss.

**Trial registration:**

The protocol for this study was registered with Clinical Trials number NCT02485210 on 30 july 2015.

## Background

Dental caries and tooth injuries are the main causes of pulp inflammation in primary teeth. In cases of irreversible pulpitis or pulp necrosis, radical endodontic treatment is indicated, the aim of which is to avoid premature tooth loss and prevent harm to the tooth bud of the permanent successor [1–4].

Chronic periapical periodontitis is the major cause of the premature loss of primary teeth and it is widely accepted that this condition is related to endodontic infection [5]. The use of handheld files and irrigating solutions with disinfectant properties is currently the most common form of endodontic treatment. However, the existence of residual bacteria in many cases can lead to further infection of the root canal, which requires re-treatment or even extraction [3, 4, 6].

Pulp therapy in primary teeth is a complex matter due to difficulties in using dental instruments, the complexity of the apical delta, the biological cycle of primary teeth, physiological resorption and rhizolysis as well as a lack of cooperation on the part of the child and the need for long appointments [3, 6–10]. In primary teeth, this procedure should allow the resorption of root structures and the filling material within the normal period, which contributes to the eruption of the permanent successor without the occurrence of pathological resorption or radiolucency in the apical or furcation regions.

Any dental procedure that eliminated pain and the continuation of the pathological process was once accepted as satisfactory, but no longer. The minimum requirement of endodontic treatment is clinical and radiographic proof of treatment success and the remission of pathological processes [ 11, 12]. The elimination of pathogenic microorganisms from the root canal system is one of the essential steps to obtaining treatment success [3, 4, 7, 9, 10, 11, 13].

Photodynamic therapy (PDT) involves the use of a photosensitizer that is activated by light at a specific wavelength in the presence of oxygen. The transference of energy from the oxygen-activated photosensitizer results in the formation of a toxic oxygen species, known as singlet oxygen or free radicals. These are highly reactive chemical species that cause damage to proteins, lipids, nucleic acid and other microbial cell components [4, 7, 13–16]. It has been established that photosensitizers have a pronounced cationic charge and bond quickly to bacterial cells. Thus, photosensitizers exhibit a high degree of selectivity, killing microorganisms rather than host cells (available from: www.consort-statement.org).

PDT may be a viable option for achieving a reduction in pathogenic microorganisms during endodontic treatment, as this method is painless, easy to administer, does not lead to microbial resistance and has no systemic effects [4, 7, 15, 16]. PDT has been widely tested for the endodontic treatment of permanent teeth, demonstrating positive results in comparison to conventional treatment [13–16] (available from: www.consort-statement.org). However, few studies have evaluated the use of this type of therapy in primary teeth.

Considering the difficulties regarding the endodontic treatment of primary teeth and the knowledge that treatment success is directly related to bacterial decontamination of the root canal system, the effects of PDT for the endodontic treatment of primary teeth should be evaluated with the aim of improving this procedure.

The aim of the proposed study is to conduct a randomized, controlled, clinical trial for the clinical and radiographic evaluation of the effectiveness of PDT during the endodontic treatment of primary teeth.

## Methods

### Ethical considerations

The proposed study will be conducted in compliance with the regulatory norms governing research involving human subjects and has received approval from the institutional review board of University of Nove de Julho (São Paulo, Brazil) under process number 832.657. Following clarifications regarding the objectives and procedures, the parents/guardians of the participants will sign a statement of informed consent authorizing the participation of their children in the study, as stipulated in Resolution 196/96 of the Brazilian National Board of Health.

### Subjects

Thirty primary anterior teeth will be selected in health male and female children with no ethnicity restriction. The teeth must be with diagnosis of dental caries or trauma with pulp envolvement. The children will be recruited at pediatric dentistry of University os Nove de Julho (São Paulo - Brasil). The parents or legal guardian will sign the statement of informed consent. Based on a previous study by Pinheiro et al. [7] for sample size calculation it was considered the standard desviation (2.41) and the diference to be detected in the median of colony forming units per milimiter (CFU/ml) before and after PDT 2.9, with significance level of 5 % and power of 95 %.

### Inclusion criteria

Children aged 3 to 6 years, with at least 1 primary anterior tooth with irreversible pulpitis or pulp necrosis due to caries or trauma suitable for restoration, and at least two thirds of the root remaining; otherwise health patients who have not been submitted to antibiotic therapy in the previous 3 months.

### Exclusion criteria

Compromised health, primary teeth with more than one third root loss, lack of internal pathological resorption, impossibility of restoration, cases of re-treatment and crypt involvement.

### Experimental groups

#### Randomization

The type of treatment will be randomly determined for each tooth using tiles on which 1 of 2 numbers is printed (Table 1):

**Table 1:** Experimental groups

Experimental groups	
Group I (*n* = 15)	Conventional endodontic treatment
Group II (*n* = 15)	Endodontic treatment with administration of PDT

*PDT* photodynamic therapy

- Number 1: conventional treatment (Group I).
- Number 2: treatment with PDT (Group II).

### Blinding procedures

The clinical and radiographic evaluations of endodontic treatment will be performed by examiners who will be blinded to the allocation of the teeth to the different groups.

### Clinical and radiographic evaluations of teeth

The clinical evaluations will be performed with the child in a dental chair under the light of the reflector, with the aid of a mouth mirror and palpation of the area around the affected tooth. During the initial exam as well as the follow-up exams at 1, 3 and 6 months after endodontic treatment, the following clinical data will be recorded: history of spontaneous pain indicative of apical periodontitis, presence of fistula or abscess, gingival swelling and pathological tooth mobility. The radiographic exams will involve the evaluation of signs of radiolucency in the periapical region and pathological root reabsorption.

The data collected during the initial exam as well as the follow-up exams at 1, 3 and 6 months after endodontic treatment and the comparison of the initial diagnostic radiographic exam to the radiographic exams at 1, 3 and 6 months after treatment will constitute the basis for the evaluation of the treatment success or failure. The radiographs will be analyzed by two experienced examiners with the aid of a negative viewing box (negatoscope). These examiners will not receive any information regarding the type of treatment each tooth received. Cases of divergence will be resolved by consensus between the two examiners [17].

The following criteria will be used for the determination of treatment success or failure:

1. Complete repair (= success)
  Clinically: absence of signs and symptoms;

Radiographically: absence of pathological root resorption, normal width of periodontal ligament space, absence of lesion development in periapical regions in cases of absence of lesion in the initial diagnostic radiograph and complete regression of lesion in cases of presence of lesion in the initial diagnostic radiograph.

2. Incomplete repair (= success)
  Clinically: absence of signs and symptoms;

Radiographically: absence of pathological root resorption and reduction lesion size in the periapical region.

3. Lack of repair (= failure)
  Clinically: signs and symptoms indicative of acute apical periodontitis;

Radiographically: presence of pathological root resorption, lesion in furcation/periapical region unchanged in size during follow-up period, increase in lesion size or the development of a new lesion [17].

All radiographs will be standardized and taken with minimal geometric distortion, which is achieved using adult periapical film (Kodak, Rochester, NY, USA) in the occlusal position and a radiographic positioner for the child in the vertical direction (modified occlusal radiograph). This procedure will allow the same position of the film at the different evaluation times and standardize the incidence of X-rays, vertical and horizontal angles and distance on all radiographs taken for the same patient [17].

The same development time, intermediate washing, fixation and final washing at all evaluation times will be used to standardize the radiograph processing. Room temperature (camara escura) will also be standardized and Kodak chemicals (Kodak, Rochester, NY, USA) will be used. Radiographic exposure time, kilovolts and milliamps of the device as well as the type of film will be standardized to obtain the most identical density and contrast possible among the radiographs [17].

### Administration of PDT

**Fig. 1:**
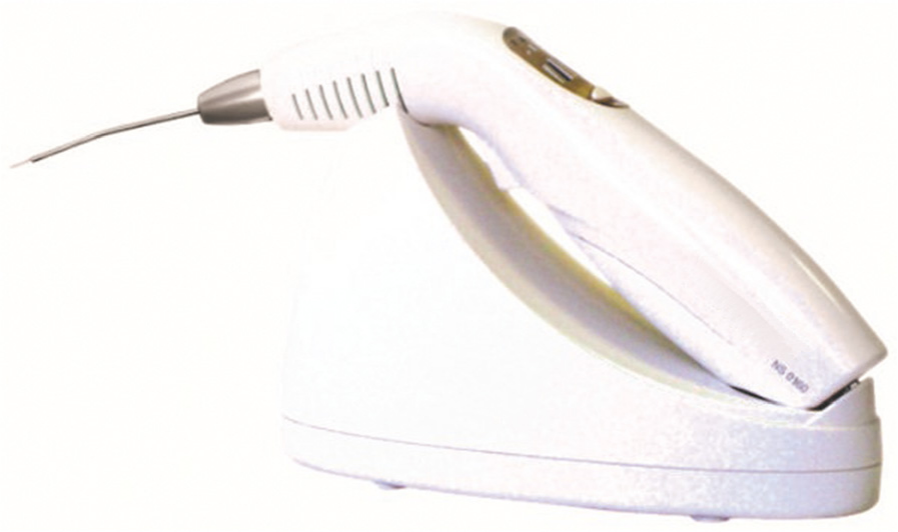
Laser Therapy XT-EC

**Fig. 2:**
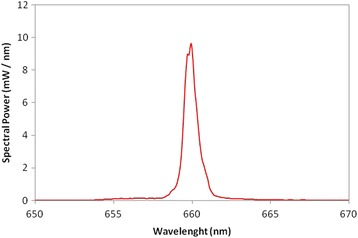
Full width at half maximum (FWHM) spectral width (nm)

The Therapy XT-EC (DMC ABC Medical and Dental Equipment, São Carlos, SP, Brazil) will be used for PDT, with laser emission in the red band (660 nm) and the narrow tip for the specific use of PDT in areas of difficult access (Figs. 1, 2). During administration, only the volunteer and operator will be present and both will wear eye protection. The active tip of the laser will be wrapped in disposable transparent plastic wrap (polyvinyl chloride, PVC) for reasons of hygiene and the avoidance of cross-contamination. The operator will be duly vested.

One session of PDT with the Chimiolux® methylene blue photosensitizer (Hypofarma, Belo Horizonte, MG, Brazil) at a concentration of 0.005 % will be performed in the interior of the root canal. The photosensitizer will be applied with a sterile paper cone for 3 minutes and the excess will be removed with an endodontic aspirator. The canal will be irradiated for 40 seconds, with the device previously calibrated to emit a wavelength at 660 nm, with radiant energy of 4 J and mean maximum power of 100 mW (Table 2). The direct contact method will be employed, based on a previous study [4, 7].

**Table 2:** Lasers parameters

Parameters	Red laser
Central wave length (nm)	659.93 nm
FWHM spectral width (nm)	0.82
Operating mode	Continuous
Mean maximum power (mW)	100
Polarization	Random
Aperture diameter (cm)	0.060
Radiance at aperture (mW/cm^2^)	38197
Beam profile	Multi-mode
Beam area (cm^2^)	1
Radiance at target (mW/cm^2^)	108
Exposure time (s)	40
Radiant exposure (J/cm^2^)	4.3
Radiant energy (J)	4.32
Number of irradiated points	1
Irradiated area (cm^2^)	1
Application technique	Contact
Number of sessions	1
Total radiant energy (J)	4.32

*FWHM* Full Widht Hay Maximum

### Interventions

All treatment procedures will be performed by a single, duly-trained operator.

### Group I (control) – conventional endodontic treatment

1. Prophylaxis with pumice stone and water and antisepsis of region with 0.12 % solution of chlorhexidine using moistened gauze.
2. Anesthesia.
3. Absolute isolation, if possible; in cases of considerable loss of remaining tooth, relative isolation using cotton rolls.
4. Opening of crown with spherical diamond tip burs (bur size compatible with tooth) (KG Sorensen – Indústria e Comércio, Cotia, SP, Brazil) and high-speed Z endodontic drill under water and air refrigeration.
5. Millimeter measurement of root canal to 1 mm short of radiographic apex.
6. Insertion of 3 sterile paper cones with diameter compatible with canal for 30 seconds for initial collection of bacterial sample and immediate placement into brain heart infusion broth.
7. Surgical chemical preparation with series of Kerr files appropriate for each case, using an initial file and an additional 2 files of larger size, with irrigation and aspiration with 1 % sodium hypochlorite (Milton’s solution) and Endo-PTC (Fórmula e Ação, São Paulo, SP, Brazil) with each change of file.
8. Insertion of 3 sterile paper cones for 30 seconds for second collection of bacterial sample and immediate placement into brain heart infusion broth.
9. Filling of root canals with calcium hydroxide (UltraCal, Ultradent, Indaiatuba, SP, Brazil); base of thin *gutta-percha* and filling with glass ionomer cement; restorative treatment performed in subsequent session.

### Group II – conventional endodontic treatment + PDT

1. Prophylaxis with pumice stone and water and antisepsis of region with 0.12 % solution of chlorhexidine using moistened gauze.
2. Anesthesia.
3. Absolute isolation, if possible; in cases of considerable loss of remaining tooth, relative isolation using cotton rolls.
4. Opening of crown with spherical diamond tip burs (bur size compatible with tooth) (KG Sorensen – Indústria e Comércio, Cotia, SP, Brazil) and high-speed Z endodontic drill under water and air refrigeration.
5. Millimeter measurement of root canal to 1 mm short of radiographic apex.
6. Insertion of 3 sterile paper cones with diameter compatible with canal for 30 seconds for initial collection of bacterial sample and immediate placement into brain heart infusion broth.
7. Surgical chemical preparation with series of Kerr files appropriate for each case, using initial file and an additional 2 files of larger size, with irrigation and aspiration with 1 % sodium hypochlorite (Milton’s solution) and Endo-PTC (Fórmula e Ação, São Paulo, SP, Brazil) with each change of file.
8. Insertion of sterile paper cone immersed in Chimiolux® methylene blue (Hypofarma, Belo Horizonte, MG, Brazil) for 3 minutes; administration of wireless Therapy XT- EC laser device (DMC ABC Medical and Dental Equipment, São Carlos, SP, Brazil) after removal of cone; energy density: 4 J/cm^2^, power: 100 mW; wavelength: 660 nm; exposure time: 40 seconds.
9. Root canal irrigated with sterile saline solution and insertion of 3 sterile paper cones for 30 seconds for second collection of bacterial sample and immediate placement in brain heart infusion broth.
10. Filling of root canals with calcium hydroxide (UltraCal, Ultradent, Indaiatuba, SP, Brazil); base of thin *gutta-percha* and filling with glass ionomer cement; restorative treatment performed in subsequent session.

**Fig. 3:**
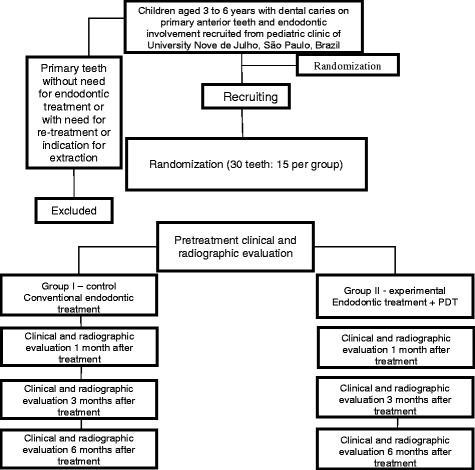
Flowchart of proposed study

The Consolidated Standards of Reporting Trials (CONSORT) [17] will be followed to ensure greater transparency and quality of the proposed randomized clinical trial (Fig. 3).

### Microbiological analysis

All samples will be collected with three paper cones, which will be inserted into the root canal before and immediately after treatment and placed into brain heart infusion broth for culturing to allow the determination of the total number of viable bacteria. The samples will be homogenized for 3 minutes in a vortex agitator, followed by dilution in 4.5 ml of peptone water of the order of 10^−6^. Three 25-μl aliquots of the decimal solution will be sewn into Petri dishes with blood agar [7]. The cultures will be incubated for 5 days at 37 °C in an 85 % nitrogen (N_2_), 10 % carbon dioxide (CO_2_) and 5 % hydrogen (H_2_) atmosphere obtained though the use of an anerobiosis generation system to allow the visualization of the total number of viable bacteria in CFU [7].

### Data organization and statistical analysis

The data will be tabulated and presented in a descriptive and analytical manner. Descriptive statistics will involve the determination of frequency, mean and standard deviation values. The Kolmogorov-Smirnov test will be used to determine the distribution of the data (normal or non-normal). Depending on the distribution, differences between groups regarding mean CFU and percentage reduction in bacteria will be determined using either the *t* test, analysis of variance (ANOVA) or the Mann-Whitney test. The Wilcoxon test will be used for comparisons before and after treatment. *P* values < 0.05 (95 % significance level) will be considered indicative of statistically significant differences. All statistical analyses will be conducted with the aid of the SPSS 21 program (SPSS Inc., Chicago, IL, USA).

## Discussion

As successful endodontic treatment is directly related to intra-canal bacterial disinfection and considering the difficult task of endodontic treatment in primary teeth, often due to difficulties in controlling young children, the internal anatomy of root canals and root resorption, the alternative of using PDT is a painless, easy-to-administer method that does not lead to microbial resistance and can assist in the achievement of successful endodontic treatment in primary teeth by eliminating the pain children can experience due to re-treatment as well as premature tooth loss.

### Trial status

The authors are currently recruiting participants. This began in September 2015 and we plan to continue until December 2015.

## Abbreviations

ANOVA: analysis of variance
CFU: colony forming units
CONSORT: Consolidated Standards of Reporting Trials
FWHM: full width at half maximum
PDT: photodynamic therapy
PVC: polyvinyl chloride
